# Estimating the effect of annual PM_2·5_ exposure on mortality in India: a difference-in-differences approach

**DOI:** 10.1016/S2542-5196(24)00248-1

**Published:** 2024-12

**Authors:** Suganthi Jaganathan, Massimo Stafoggia, Ajit Rajiva, Siddhartha Mandal, Shweta Dixit, Jeroen de Bont, Gregory A Wellenius, Kevin J Lane, Amruta Nori-Sarma, Itai Kloog, Dorairaj Prabhakaran, Poornima Prabhakaran, Joel Schwartz, Petter Ljungman

**Affiliations:** Institute of Environmental Medicine, Karolinska Institutet, Stockholm, Sweden (S Jaganathan MPH, M Stafoggia PhD, J de Bont PhD, P Ljungman PhD); Centre for Health Analytics Research and Trends, Ashoka University, Sonipat, India (S Jaganathan, A Rajiva MESc, S Mandal PhD, S Dixit PhD, P Prabhakaran PhD); Centre for Chronic Disease Control, New Delhi, India (S Jaganathan, A Rajiva, S Mandal, S Dixit, Prof D Prabhakaran DM, P Prabhakaran); Department of Epidemiology, Lazio Region Health Service/ASL Roma 1, Rome, Italy (M Stafoggia); Department of Geography and Environment, Faculty of Humanities and Social Sciences, Ben-Gurion University of the Negev, Beer-Sheva, Israel (A Rajiva, Prof Itai Kloog PhD); Department of Environmental Health, School of Public Health, Boston University, Boston, MA, USA (Prof G A Wellenius ScD, K J Lane PhD, A Nori-Sarma PhD); Department of Environmental Medicine and Public Health, Icahn School of Medicine at Mount Sinai, New York, NY, USA (Prof I Kloog); Public Health Foundation of India, Gurugram, India (Prof D Prabhakaran); Department of Environmental Health, Harvard T H Chan School of Public Health, Boston, MA, USA (Prof J Schwartz PhD); Department of Cardiology, Danderyd University Hospital, Danderyd, Sweden (P Ljungman)

## Abstract

**Background:**

In 2019, the Global Burden of Diseases, Injuries, and Risk Factors Study attributed 0·98 million deaths to ambient air pollution in India based on potentially inappropriate exposure–response functions from countries with low air pollution levels. Instead, using data from India, we investigated long-term exposure to PM_2·5_ and all-cause mortality with a causal inference method.

**Methods:**

We collected national counts of annual mortality from 2009 to 2019 from the Civil Registration System at the district level to calculate annual district-level mortality rate as our main outcome and obtained annual PM_2·5_ concentrations from a high-resolution spatiotemporal model. We applied an extended version of the difference-in-differences design by use of generalised additive models with quasi-Poisson distribution, including indicator variables and separate time trends for spatial administrative divisions. PM_2·5_ concentrations obtained at 1 km × 1 km spatial resolution across the country were used to calculate annual district-level mean PM_2·5_ concentrations. Similarly, we collected confounders at the district level, such as mean and SD of quarterly temperatures, gross domestic product per capita, population aged 60 years or older, clean cooking fuel usage, literacy in women, and median age. The spatial unit of analysis was administrative division.

**Findings:**

The annual median population-weighted PM_2·5_ was 38·9 μg/m^3^ (5–95th percentile 19·7–71·8 μg/m^3^). The full population lived in areas with PM_2·5_ concentrations exceeding the 5 μg/m^3^ annual mean recommended in the WHO guidelines, and 1·1 billion of 1·4 billion (81·9% of the total population) lived in areas above the Indian National Ambient Air Quality Standards for annual mean PM_2·5_ not exceeding 40 μg/m^3^. A 10 μg/m^3^ increase in annual PM_2·5_ concentration was associated with an 8·6% (95% CI 6·4–10·8) higher annual mortality. Based on the Indian National Ambient Air Quality Standards, a total of 3·8 million (95% CI 2·9–4·9) deaths between 2009 and 2019 were attributable to PM_2·5_, amounting to 5·0% (3·8–6·4) of total mortality. Based on the WHO guidelines, a total of 16·6 million (13·0–21·8) deaths were attributable to PM_2·5_, amounting to 24·9% (19·5–32·5) of total mortality.

**Interpretation:**

Our difference-in-differences approach allowed us to assess the full extent of registered deaths in the most populated country in the world, which has high levels of air pollution. We provide new evidence of increased mortality risk from long-term PM_2·5_, which emphasises the need for tighter regulatory standards to potentially substantially reduce mortality across India.

**Funding:**

Swedish Research Council for Sustainable Development.

## Introduction

Long-term exposure to PM_2·5_ has been associated with many health effects, including all-cause mortality. The Global Burden of Diseases, Injuries, and Risk Factors Study (GBD) attributed 4·4 million (95% CI 3·5–4·8) deaths in 2019 to long-term ambient PM_2·5_ exposure,^1^ whereas the Global Exposure Mortality Model predicted a higher estimate of 8·9 million (95% CI 7·5–10·3) deaths in 2015.^2^ Few studies have focused on the link between long-term PM_2·5_ exposure and mortality in the Asia–Pacific region, and existing studies are cohort studies mainly from Australia, mainland China, Hong Kong, Taiwan, and South Korea.^3^ Despite very high air pollution and a large population exposed in India there are scarce data linking long-term PM_2·5_ exposure and mortality to adequately guide public policy. GBD 2019 estimated that 0·98 million deaths in India were attributable to ambient air pollution, corresponding to an attributable fraction of 10·4%.^4^ A few other studies from India have conducted health impact assessment analyses for cause-specific^5,6^ and premature mortality^7,8^ in relation to ambient PM_2*·*5_ exposure, adapting the exposure–response function from GBD. The reliance on exposure–response functions that are mostly derived from countries with lower air pollution levels than India poorly represents Indian exposure levels and specific source contribution and therefore exposure–response functions are not necessarily comparable in terms of baseline risks or population characteristics.

In India, people living in urban and several rural areas are exposed to high PM_2·5_ concentrations throughout the year.^9^ Population-weighted mean PM_2·5_ exposure (2000–19) across India was reported as 57·3 μg/m^3^ (5–95th percentile range 16·8–86·9), with higher concentrations observed from 2010 to 2019.^10^ WHO revised the air quality guidelines for PM_2·5_ concentrations in 2021 based on mounting evidence of major health effects even at low exposure concentrations, resulting in stricter recommendations, going from not exceeding 10 μg/m^3^ to not exceeding 5 μg/m^3^ annual mean PM_2·5_ concentration.^11^ However, current Indian National Ambient Air Quality Standards (NAAQS) are less than or equal to 40 μg/m^3^ for annual mean PM_2·5_ concentration, which is eight times higher than the WHO guidelines. Hence, providing evidence from local studies to adequately assess the health risks from air pollution in India is key to informing and developing public health policy, including the NAAQS.

India’s Ministry of Environment, Forest, and Climate Change has been making efforts since 2017 to address air pollution with the National Clean Air Programme, which focuses on regulations, policies, and programmes to improve air quality in 132 cities.^12^ Although this programme represents a positive step, PM_2·5_ concentrations have increased from 2010 to 2019.^5^ Moreover, focusing on only local control efforts in cities might be inadequate since the settling time of PM_2·5_ particles is a week or more, during which these particles can travel 1000 km or further.^13–15^

Providing robust unbiased evidence by use of causal modelling offers important opportunities in observational settings where experimental studies are not feasible for exposures, such as for air pollution.^16,17^ The major advantage is that this type of modelling mimics experimental studies, deriving causal associations while exploiting the full potential of large observational administrative data. The difference-in-differences method is one such quasi-experimental approach that controls for unmeasured confounders by design,^18,19^ by comparing the changes in outcomes over time between an exposed group and unexposed or less exposed group.^17^

Given the complex and nationwide high air pollution exposure and scarcity of long-term exposure studies in India, we estimated the effect of annual exposure to PM_2·5_ on annual all-cause mortality over a decade (2009–19) using the difference-in-differences approach. Furthermore, we derived the exposure–response function from the Indian context and assessed the public health impact by calculating the attributable fraction in relation to WHO guidelines and the Indian NAAQS.

## Methods

### Study area and mortality assessment

For each of the 655 Indian districts, and each year from 2009 to 2019, we retrieved the annual all-cause mortality data from the Vital Statistics Division, Civil Registration System Section, New Delhi, India, using 100% of all reported deaths in the country.^20^ For each district and year, we then obtained data on the resident population density (Columbia University Socio Economic Data and Applications Center [SEDAC], Palisades, NY, USA),^21^ which was used to calculate annual mortality rates by district. We also collected state-level completeness of death registration, reported by the Civil Registration System, Delhi, India, defined as the proportion of registered deaths to total deaths estimated through the Sample Registration System for a given year.^20^

### Covariate information

The covariates were selected based on the previous literature and directed acyclic graphs (appendix p 2). The covariates were available at different spatial and temporal resolution (appendix pp 2-3). We harmonised all covariates by aggregating at district level. The following covariates were available at 1 km^2^ spatial resolution: population density, gross domestic product (GDP) per capita, and quarterly mean and SD of temperature. The covariates available at district level were the proportion of the population aged 60 years or older, proportion of households using clean fuel for cooking (%), and proportion of women who are literate. The following variables were available at state level: median age and completeness of death registration, with an assumption that all districts within each state had same values.

Briefly, we used population density (per 1 km^2^) from the SEDAC^21^ available at three timepoints (ie, 2010, 2015, and 2020) and linearly interpolated for the in-between years. We used gridded data on GDP,^22^ which were available annually, as an indicator of district-level socioeconomic status for 2009–19. Quarterly mean temperatures and their SDs were computed from monthly temperatures, weighted by population, and the mean was calculated at district level from the European Centre for Medium Range Weather Forecast (Reading, UK). Proportion of the population aged 60 years or older was extracted from the 2011 census and was considered as constant. As an indicator for indoor air pollution, we used the district-level proportion of households using clean fuel for cooking, taken from the 2011 census^23^ and the National Family Health Survey^24,25^ (wave 4 [2014–15] and wave 5 [2019–20]), with linear interpolation for the in-between years. Literacy rate among women was collected from National Family Health Survey waves 4 and 5, with linear interpolation for the in-between years. Data on state-level median age for 2011, 2016, and 2021 were available from the National Commission on Population.^26^ We assumed all districts within each state had same median age, and data were linearly interpolated for the in-between years.^26^

### Exposure assessment

We obtained annual mean PM_2·5_ concentrations at 1 km × 1 km spatial resolution across India using an ensemble averaging approach for the study period. The detailed explanation of the modelling approach is published elsewhere.^27^ Briefly, we collected ground-monitoring-based observations of daily mean PM_2·5_ and PM_10_ across 1056 locations and an extensive set of predictors encompassing satellite-based observations, meteorology, land-use patterns, emissions inventories at a 25 km spatial resolution,^28^ and reanalysis-based data. Using a cross-validation approach by leaving out 20% of the monitors, we trained four machine learning methods (ie, deep learning, random forests, gradient boosting, and extreme gradient boosting) on the training data, regressing the ground-monitoring data against predictors to calibrate them. Each algorithm was trained with an internal hyperparameter tuning with hyperparameter choices mentioned in the supplementary material of Mandal and colleagues’ study.^27^ The optimised models were then implemented on the left-out validation data to obtain learner-specific predictions and externally validate these models on data from the 20% (211 of 1056 monitoring stations) of monitoring data that was left out. Individually, the machine learning algorithms showed different prediction accuracies according to temporal period and spatial characteristics. The ensemble averaging was done to borrow strength across the different machine learning algorithms and was done by use of a Gaussian process-based model (including elevation and land-use features) to combine the predictions from the four different algorithms into one final prediction for each grid-day combination. This method allowed us to obtain PM_2·5_ exposures in areas with no monitoring data across time. Cross-validated predictions for annual means were very high (*R*^2^=0·94, mean absolute error=8·8 μg/m^3^) across the country. For this study, we aggregated the district-level PM_2·5_ concentrations (all 1 km × 1 km grid cells contained within each district) annually for the entire study period (ie, 2009–19) and weighted by its population. Population-weighted means were used to provide a more accurate representation of the actual exposure for the population.

### Statistical analysis using the difference-in-differences approach

**Figure 1:**
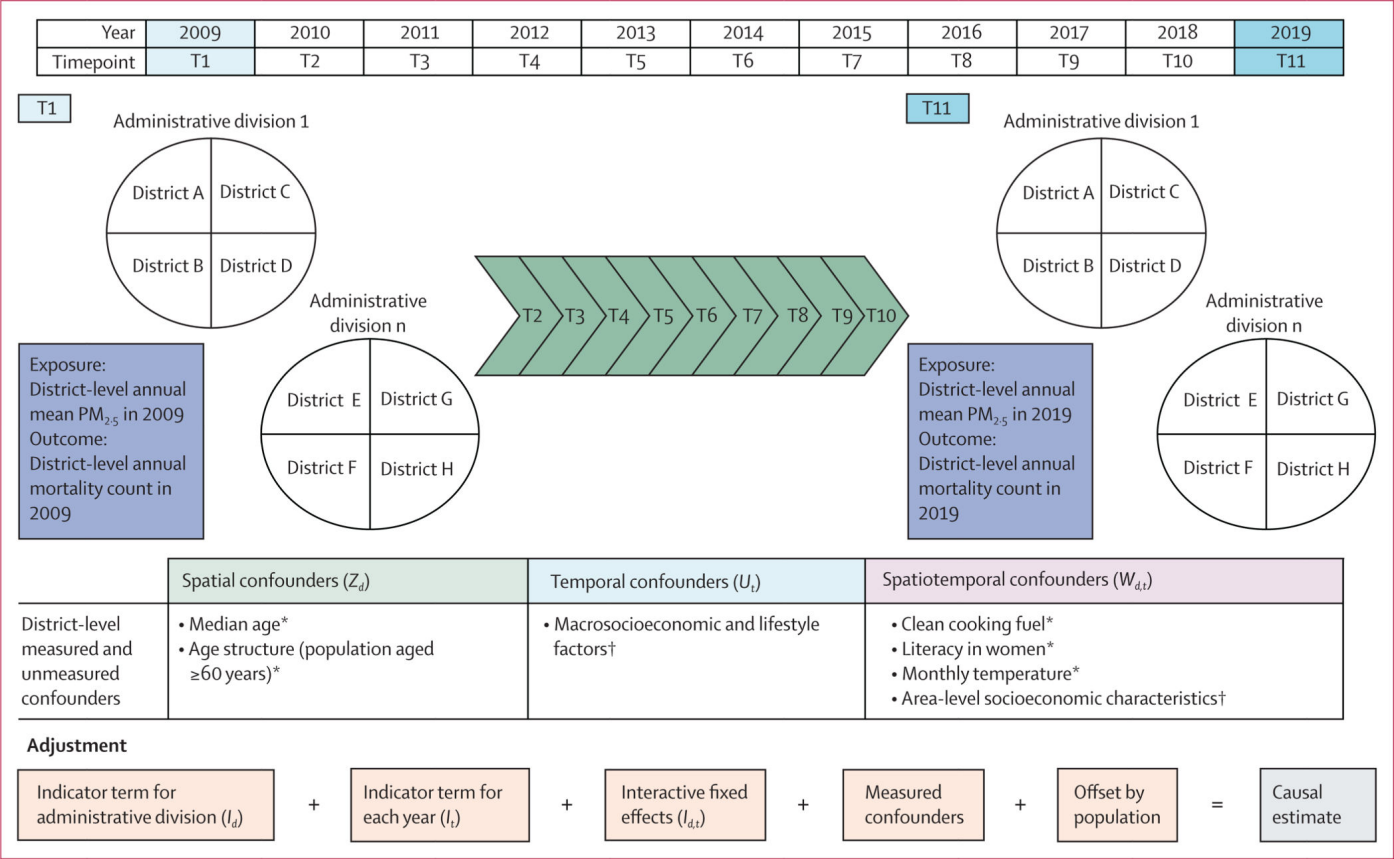
A conceptual framework for studying the effect of PM_2·5_ and all-cause mortality using difference-in-differences approach *Measured confounders. †Unmeasured confounders.

Using the potential outcomes framework (ie, Rubin’s causal model^17^), let $Y_{d,t}^{A=a}$ be the potential outcome (ie, aggregated number of deaths) in the population of district d if exposed to A=a in year t, and let $Y_{d,t}^{A=a}$ be the potential outcome under the alternative exposure *á*. We aimed to estimate $E(Y_{d,t}^{A=a})/E(Y_{d,t}^{A=a'})$, the causal effect of the change in exposure on the outcome. By generalising the previous concept to the case of a continuous exposure a, and following the notation in Wang and colleagues,^29^ we assume that the potential outcome depends on exposure and predictors in the following manner: $\ln\left(E\left[Y_{d,t}^{a}\right]\right)=\beta_{0}+\beta_{1}\alpha +\beta_{2}Z_{d}+\beta_{3}U_{t}+\beta_{4}W_{d,t}+\ln\left(P_{d,t}\right)$, where $Z_{d}$ represents spatial covariates (ie, characteristics that vary among administrative divisions but not over the time, such as age structure and macrosocioeconomic indices that are unlikely to vary in the short-term span of a few years); $U_{t}$ represents temporal covariates (ie, characteristics that vary over time but uniformly across administrative divisions, such as secular trends in mortality rates and macrosocioeconomic or lifestyle gradients potentially varying within a decade, but similarly across the country); $W_{d,t}$ represents covariates that vary over time differently across administrative divisions (eg, meteorological patterns and area-level socioeconomic characteristics); ln $\left(P_{d,t}\right)$ is an offset term representing the natural log of the population in administrative division d and year t, and serves the purpose of modelling mortality rates, rather than counts; and α represents the annual mean concentration in PM_2·5_ for that specific district and year (figure 1).

In the above conceptual model, by adding suitable indicator variables for space (ie, administrative divisions), time (ie, years), and their interaction, we automatically control for all potential (ie, known and unknown, measured and unmeasured) covariates changing differently over time across administrative divisions and covarying with air pollution concentrations. Since our spatial units of analysis are districts, and not divisions, what remains to be adjusted for are spatiotemporal covariates $W_{d,t}$ varying differently over time across districts in the same division and covarying with annual district-specific air pollution levels.

We converted the previous conceptual model by fitting the following quasi-Poisson generalised additive model:

$$g\left(E\left[Y_{d,t}\right]\right)=\ln\left(P_{d,t}\right)+\beta_{0}+\beta_{1}PM_{2.5}\;\;+\text{ ns(time,by=administrative division)}\;\;+\text{ covariates}$$

where g(.) denotes the link logarithmic function; $E\left[Y_{d,t}\right]$ is the expected value of the mortality count in district d on year t; $P_{d,t}$ is the population for the same district and year, serving as offset term; $\beta_{0}$ is the model intercept; and $\beta_{1}$ is the regression coefficient of PM_2·5_ representing the causal effect of the exposure on the outcome. ns(time,by=administrative division) represents the interactive fixed effects component of the model, namely a separate time trend (modelled with natural splines with 3 df) for each administrative division, aimed at capturing division-specific spatial (*Z*), temporal (*U*), and spatiotemporal (*W*) confounders. Covariates were meant to adjust for potential residual confounding induced by district-level covariates and include season-specific or quarterly mean and SD of air temperature, proportion of the population aged 60 years or older, GDP per capita in US dollars, proportion of households with clean cooking fuel, proportion of women who are literate, and median age.

Finally, we relaxed the assumption of linearity in the PM_2·5_–mortality association by replacing the linear term with a natural spline with 4 df to explore the shape of exposure–response function.

### Sensitivity analysis

We implemented sensitivity models aimed at checking the robustness of our main findings. Since we noted that death reporting varies widely across the country, our first sensitivity model calibrated for completeness of death at the state level by multiplying the actual number of reported deaths with completeness of death reporting expressed as a percentage at state level. In the next sensitivity analysis, we excluded extreme levels of PM_2·5_ concentrations (ie, 1st or above the 99th percentiles) to explore the association when extreme values were excluded. Furthermore, we examined for the lagged exposures lag 1, meaning that we shifted the exposure assignment to the preceding year (eg, used annual mean PM_2·5_ concentration of 2008 for analysis of annual district-level mortality in 2e009), and combined lag 0 and lag 1, wherein we assigned a 2-year mean (eg, assigning mean PM_2·5_ 2008–09 to mortality counts in 2009).

### Attributable mortality

To estimate the attributable deaths and fractions, we applied the coefficient of PM_2·5_ from the final model, using two counterfactual concentrations: the Indian NAAQS (ie, ≤40 μg/m^3^) and the WHO guidelines (ie, ≤5 μg/m^3^) for annual means. We used the Indian NAAQS to motivate policy makers regarding the current guidelines and aid in understanding the need for revising guidelines, without necessarily implying that these two concentrations are safe.

Attributable mortality was calculated as,

$$\text{M}(\text{\#})=\frac{e^{\beta \text{Δ}X}-1}{e^{\beta \text{Δ}X}}N_{di}$$

where β is the coefficient from the final model per 1 μg/m^3^ increase in annual mean PM_2·5_ exposure, Δ*X* is the difference between the observed PM_2·5_ concentration and the threshold (ie, Indian NAAQS or WHO guidelines) at the district level, and $N_{di}$ is the total number of deaths for each district (*d*) for a given year (*i*).

All statistical analyses were conducted in R (version 4.1.2) and the following packages were used: splines, gnm, mgcv, and ggplot2 for the figures. Maps were created using QGIS (version 3.28). This work was approved by the Health Ministry Screening Committee at the Indian Council of Medical Research and the ethical committee at the Public Health Foundation of India.

### Role of the funding source

The funder of the study had no role in study design, data collection, data analysis, data interpretation, or writing of the report.

## Results

**Figure 2:**
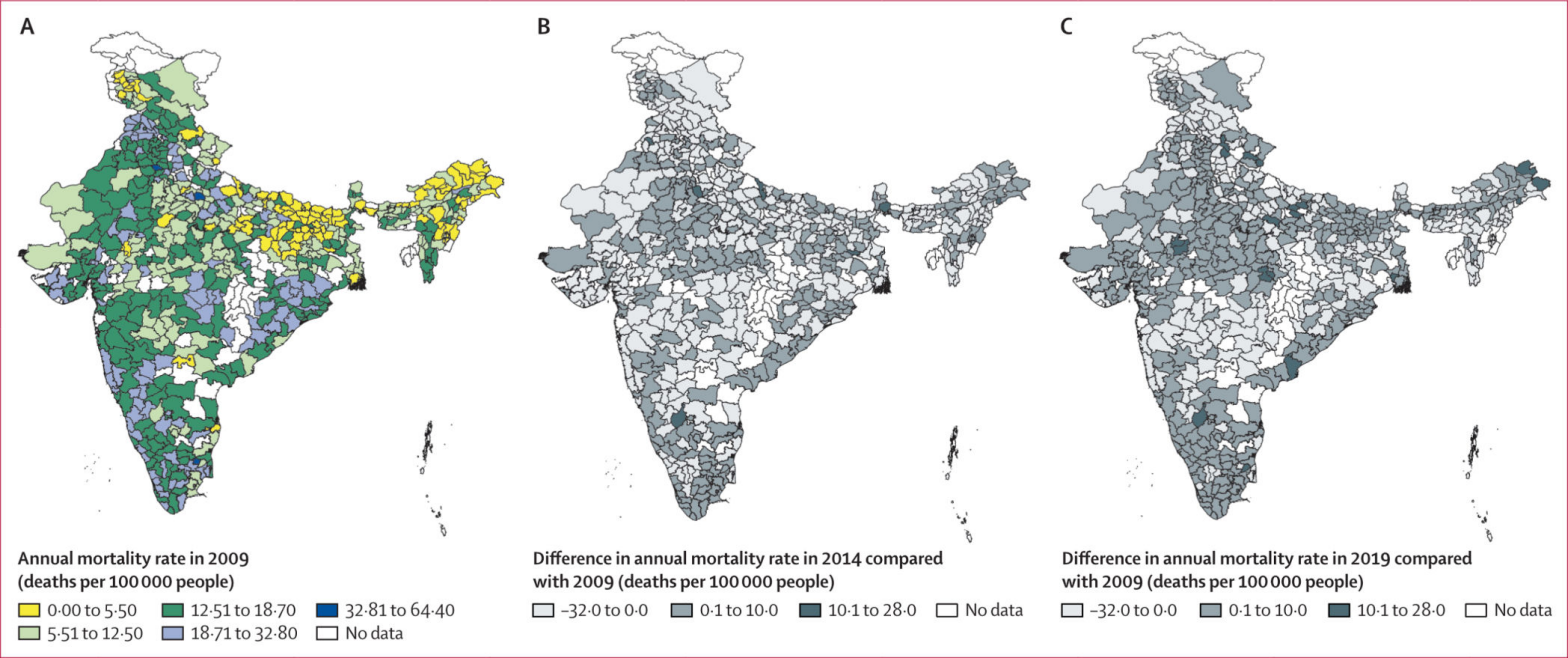
Mortality across India (A) Annual mortality rate in 2009. (B) Differences in mortality rate in 2014 compared with 2009. (C) Differences in mortality rate in 2019 compared with 2009.

**Figure 3:**
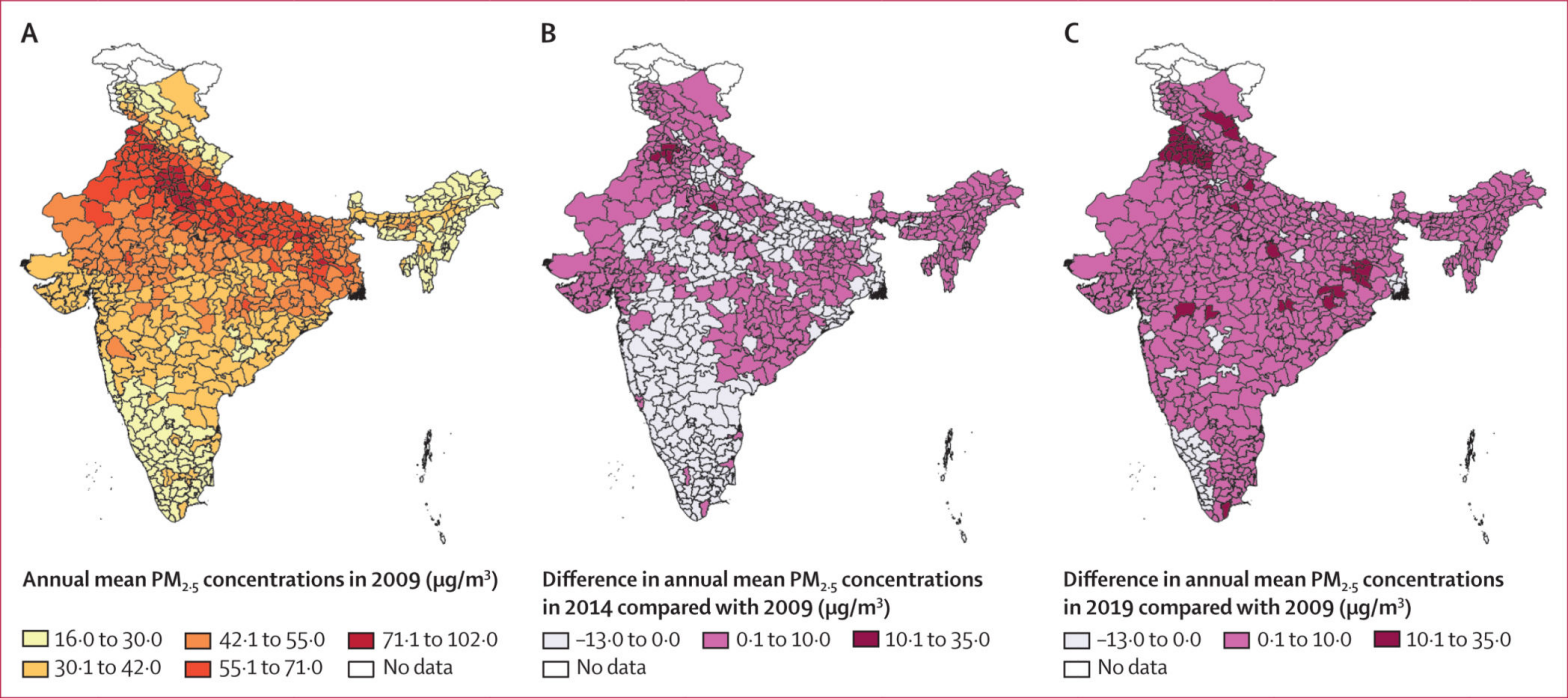
PM_2·5_ concentrations across India (A) Annual mean concentrations of PM_2·5_ in 2009. (B) Differences in annual concentrations in 2014 compared with 2009. (C) Differences in annual concentrations in 2019 compared with 2009.

Descriptive information of the study area is provided in table 1. The entire 1·4 billion people in the Indian population lived in areas with PM_2·5_ concentrations above that recommended by the WHO guidelines, and 1·1 billion of 1·4 billion (81·9% of the total population) lived in areas above the Indian NAAQS. The national population-weighted median PM_2·5_ per district was 38·9 μg/m^3^ (5–95th percentile 19·7–71·8 μg/m^3^) for the whole study period. The range of PM_2·5_ exposure was quite large across the years, with the minimum PM_2·5_ concentration of 11·2 μg/m^3^ observed in Lower Subansiri district, Arunachal Pradesh, in 2019 and the maximum of 119·0 μg/m^3^ observed in Ghaziabad, Uttar Pradesh, and Delhi in 2016 (data for each district are not shown). Reported annual mortality ranged from 4·5 million in 2009 to 7·3 million in 2019. Across the states, the annual mean completeness of death registration ranged from 30·2% to 100·0%, with ten states and three union territories presenting more than 90% death registration throughout the study period. Figure 2 shows the annual mortality rates across three time periods, and figure 3 shows the annual mean concentrations of PM_2·5_ across three time periods.

**Figure 4:**
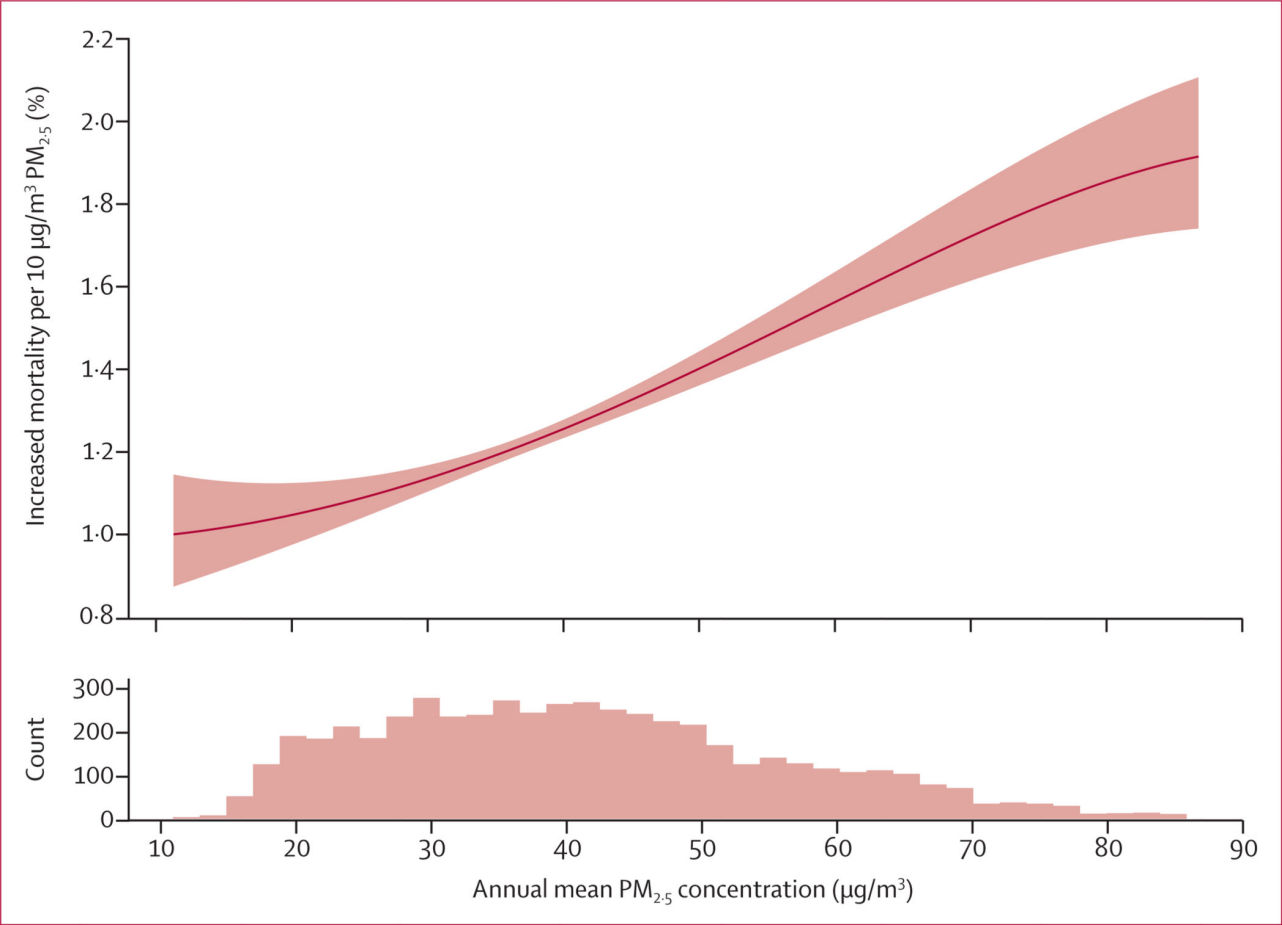
Exposure–response function for long-term exposure to PM_2·5_ and annual mortality PM_2·5_ concentration is shown up to the 99th percentile.

In the crude model, we observed a 14·1% (95% CI 11·8–16·5) increased risk for annual all-cause mortality with every 10 μg/m^3^ increase in annual average PM_2·5_. In the final (difference-in-differences) model, with separate time trends per administrative division, we estimated an increase in annual all-cause mortality rates by 8·6% (6·4–10·8) per 10 μg/m^3^ PM_2·5_. Estimates for each modelling step are provided in the appendix (p 3). In figure 4, we show the non-linear exposure–response function for annual all-cause mortality and annual mean exposure to PM_2·5._

Results were robust to several sensitivity analyses, including adjustment for death registration and removal of extreme values of PM_2·5_ (1st and 99th percentiles; appendix p 3). We performed an additional analysis using lagged exposure (lag1; ie, assigned PM_2·5_ exposure from 2008 to people who died in 2009). With lag1 exposure, we estimated an increase in annual all-cause mortality rates by 8·5% (95% CI 6·3–10·8) per 10 μg/m^3^ PM_2·5_, which is very similar to the final model results. With combined lag0 and lag1 exposure, we estimated an increase in annual all-cause mortality rates by 9·3% (6·9–11·7) per 10 μg/m^3^ PM_2·5_.

In table 2, the attributable deaths and fractions are presented for the Indian NAAQS of an annual mean of 40 μg/m^3^ or lower and the WHO ambient air quality guideline of not exceeding 5 μg/m^3^. As expected, in comparison with the WHO guidelines, we showed a significant mortality burden, but we also observed a marked number of attributable deaths using the eight-fold higher Indian NAAQS. For instance, we estimate that about 1·8 million (95% CI 1·4–2·4) deaths were attributed to PM_2·5_ in 2019 using the revised WHO guidelines (appendix p 4). Yearly attributable deaths and fractions are provided in the appendix (p 4).

## Discussion

In this comprehensive nationwide study from 2009 to 2019 in India, we investigated the association between long-term exposure to PM_2·5_ and annual mortality in the general population of India using a difference-in-differences approach. We estimated that every 10 μg/m^3^ increment in PM_2·5_ was associated with an 8·6% increase in risk for all-cause mortality, with annual average PM_2·5_ concentrations in Indian districts ranging between 11·2 μg/m^3^ and 119·0 μg/m^3^.

Our difference-in-differences methodology, using granular pollution and mortality data, potentially provides a more accurate estimate of PM_2·5_-related mortality than previous work and, since the necessary assumptions of the approach are met and most potential confounders are adjusted for by design, the chances are minimised for residual confounding from omitted covariates in our estimates. Using the modified difference-in-differences approach, we captured changes in mortality across timepoints in each location and between locations, justifying the definition of quasi-experimental design given to this technique. The difference-in-differences approach controlled for confounders at the spatial unit of analysis, in our case an administrative division, representing a population under study in which common confounding variables, such as age, sex, and smoking, are normally distributed and stable over time, whether they are measured or not. Therefore, all of the time-invariant confounders (ie, both individual and area level) are removed by design, and the method focuses on the time-varying confounders. Covariates that differ by administrative division are also controlled. In our analysis, we controlled for potential confounding by the district-level variables, such as socioeconomic status (GDP per capita), proportion of the population aged 60 years or older, population density, proportion of women who are literate, median age, and mean and SD of quarterly temperature. Having separate splines of time for each administrative division allowed for different time trends in outcome among different administrative divisions during the study period. Using separate splines for each year across divisions enabled our analysis to focus on year-to-year changes around those time trends in exposure and mortality counts in each year and division. Notably, the analysis also adjusted for quarterly temperatures and their SDs and indoor air pollution, both considered as the main confounders. The results from the final model were extremely flexible in capturing confounding from spatiotemporal covariates, plus residual confounding from unmeasured (known or unknown) covariates changing differently over time across administrative divisions, therefore relaxing the parallel-trend assumption.

Only one study from India has investigated associations between long-term exposure to PM_2·5_ and total mortality and reported null results using a low resolution PM_2·5_ model in a subsample of the population aged 15–69 years.^30^ Other studies examining the association between ambient PM_2·5_ exposure and mortality in India did not use causal methods and hence are not directly comparable to our results.^5–8^ Chowdhury and colleagues^5^ reported that non-linear power law estimation predicted 0·4 million annual deaths, whereas the GBD’s integrated response function predicted 0·8 million annual deaths in India after baseline mortality adjustment. The authors acknowledged the need for developing India-specific risk functions to settle these differences in risk estimation. Our estimate of 1·5 million (95% CI 1·1–1·9) deaths annually due to ambient air pollution is slightly higher than the GBD’s integrated exposure–response function. The health impact assessment study by Saini and colleagues^6^ reported a reduction of approximately 18% in premature mortality in million-plus cities by adhering to NAAQS (ie, ≤40 μg/m^3^) and a 70% reduction by adhering to older WHO guidelines (ie, not exceeding 10 μg/m^3^) for annual PM_2·5_ concentration. These results were widely different from our study, in which we estimated an attributable fraction of only 5·0% over the Indian NAAQS-recommended concentration and 24·9% over the 2021 WHO-recommended concentration, and the GBD study. Another health impact assessment study by Ghude and colleagues^7^ estimated 570 000 (95% CI 320 000–730 000) premature mortalities in 2011 from PM_2·5_ exposure, but it is not clear what threshold values were used in this estimation. Finally, a study by Jia and colleagues^8^ in Indian states reported that the number of total deaths increased from 580 000 in 1998 to 700 000 in 2015 due to ambient PM_2·5_. In summary, we believe that our study provides the most accurate exposure–response function and health impact assessment in India to date based on causal estimations from a state-of-the-art comprehensive exposure assessment and nationwide mortality data collected in India.

Most other studies, apart from a study from Japan,^31^ have consistently reported increased risks. When comparing our results with the previous literature, especially considering studies conducted in areas with higher PM_2·5_ exposures, our estimates per 10 μg/m^3^ increase in PM_2·5_ are generally in line with exposure–response estimates from meta-analyses. An 8·0% increased mortality per 10 μg/m^3^ was reported in a large meta-analysis that included 104 cohort studies and three case-control studies conducted in Europe, North America, and parts of Asia (ie, China, South Korea, Taiwan, and Japan).^32^ This result is consistent with another meta-analysis that included studies from North America, Europe, China, Taiwan, and Japan and reported lower exposure response functions at higher levels of PM_2·5_, with an 8·2% increased mortality in studies with mean exposure of 30 μg/m^3^.^33^ A systematic review including seven studies from Asia–Pacific with results from the general population mixed with subpopulations of patients with kidney disease and tuberculosis and older people reported 3·0–11·0% increased mortality per 10 μg/m^3^ PM_2·5_ at mean exposures of 4·5–50·0 μg/m^3^.^3^ Higher effect estimates have been reported in studies with lower mean exposure ranges, such as a study from New Jersey, USA, with a mean PM_2·5_ exposure of 10·3 μg/m^3^ that reported a 15·5% (95% CI 0·8–32·3) increased risk of mortality using a difference-indifferences approach. Similarly, the Harvard Six Cities study had a mean exposure of 17·9 μg/m^3^ and reported a 12·7% (4·3–21·9) increased risk of mortality.^34^ Nevertheless, there might be other possible reasons why our estimates are in the lower interval of effect. One putative reason that we considered was the influence of the differing completeness of registered deaths across states.^35^ To address this issue, in sensitivity analysis, we calibrated the reported mortality by the registration completeness and found similar results to the final model, indicating little effect of differential misclassification of deaths. Another reason could be the relatively young population of India (ie, mean age 28·7 years) compared with other countries, such as China (ie, mean age 38·4 years).^36^

We estimated that 1·5 million (95% CI 1·1–1·9) deaths occurred annually due to long-term exposure to PM_2·5_ in India every year in excess of the 5 μg/m^3^ that is recommended by the WHO ambient air quality guidelines. Although the WHO recommendation is substantially lower than the current air pollution levels in India, even when considering the Indian NAAQS the 0·3 million (95% CI 0·2–0·4) annual attributable deaths across the country is still alarmingly high. Globally attributable annual premature mortality due to ambient PM_2·5_ exposure has been estimated to range between 4·4 million and 8·9 million from GBD and global exposure mortality model analyses, respectively.^1,2^ Our analysis estimated that about 1·8 million (95% CI 1·4–2·4) deaths (appendix p 4) in India alone were attributable to PM_2·5_ in 2019 using the revised WHO guidelines, which is twice the GBD estimate of 0·9 million (0·7–1·1) deaths from the same year. The GBD model used an integrated exposure–response function derived from a multitude of research studies conducted in low-exposure settings and used environmental tobacco smoke studies as surrogates for high exposure. However, subsequent meta-analyses that included studies with high air pollution concentrations concluded that the GBD exposure–response function underestimated risk at high exposures, such as in Asia, which is also confirmed by our results.^33^

To our knowledge, this is the first study to apply a causal methodology to assess long-term PM_2·5_ exposure and all-cause mortality and address confounding by design in India. Our study made efficient use of a very large national dataset with 11 years of mortality data and could circumvent problems with the incomplete death registration levels to produce scientifically sound estimates. We adjusted for the potential contribution of indoor air pollution to ambient air pollution in our models. Furthermore, we used a robust state-of-the-art spatiotemporal exposure assessment model. Hence the exposure–response function derived from this study is adequately applicable to the Indian setting.

Despite the strengths of this study, we acknowledge several limitations. First, the outcome data on age-specific and sex-specific mortality were not available, hence stratified analysis to identify vulnerable groups was not in the scope of this study.^35^ Second, completeness of death registration across the country varied, with some states having lower registration levels than others, and we addressed these differences in a sensitivity analysis, without notable deviations from our main results.^35^ Third, although the exposure model was available at a fine spatiotemporal scale, we had to aggregate the exposures at the district level to match the spatial scale of outcome data. We observed the differences in the estimated causal effect across the administrative divisions, which could be attributed to different sources of air pollution. However, our analyses were focused on the overall effect of PM_2·5_ exposure on all-cause mortality, and we note this inability to address source-specific effects of PM_2·5_ as one of the limitations of the study. We address the different sources to some extent in our PM_2·5_ exposure model by incorporating different sources from the emission inventories^27,28^ at a 25 km spatial resolution. Fourth, some of the covariates, such as population counts, literacy among women, use of clean cooking fuel, and population aged 60 years or older were not available at all timepoints, hence these variables were imputed either linearly or considered as constant. We acknowledge that we were unable to account for other pollutants, such as NO_2_ and O_3_, that could have provided a more complete picture to our analyses by isolating the effects of PM_2·5_ from other pollutants. Our results might therefore carry some of the risk, including potential interactions, associated with other pollutants in addition to the PM_2·5_ effect, yet most of the current literature indicates that long-term exposure to PM_2·5_ is the main health hazard in air pollution.^32,37,38^ We strongly believe that disentangling the effects of PM_2·5_ from those of other pollutants should be a research priority in India and elsewhere. Finally, despite the strengths of the interactive, fixed-effects, difference-in-differences design, our analysis was still ecological in nature, as we had access only to aggregated data on the outcome and matching exposure and could not adequately explore the role of individual characteristics. In this regard, we cannot entirely rule out residual confounding induced by our ecological design. However, we believe residual confounding to be unlikely because, as explained by Lu and Zeger,^39^ when a model of aggregated counts can be derived from an individual risk model and the exposure is common across individuals, such assignment introduces Berkson error in exposure assessment without inducing any bias in the effect estimates, but might widen the confidence intervals.^39^ In all, these limitations point to the importance of future corroborating work with individual-level data and inclusion of data on additional air pollutants in high-exposure settings, such as India.

Using a difference-in-differences approach that allowed us to harness the full extent of registered deaths in one of the most populated and polluted countries in the world, we provided new evidence of increased risk of all-cause mortality from long-term exposure to PM_2·5_ in India. Our results indicated that previous data of disease burden from ambient PM_2·5_ exposure in India are considerably underestimated, stressing the imperative to make progress rapidly and comprehensively towards reducing the population exposure across India.

## Supplementary Material

Supplement file

## Figures and Tables

**Table 1:** Descriptive information across the study period, by percentile of PM_2·5_ concentration, 2009–19

	Up to 50th percentile (≤39·2 μg/m^3^)	50–99th percentile (39·3–85·8 μg/m^3^)	Over 99th percentile (>85·8 μg/m^3^)

Number of states or union territories,* n	26	18	3
Number of divisions,* n	68	89	7
Number of districts, n	301	323	7
Population affected, %	16·4% (5·9–39·1)	27·4% (18·0–43·5)	56·2% (37·6–76·1)
All-cause mortality rate per 100 000	14·2 (9·0–19·1)	13·3 (7·5–17·1)	12·3 (8·1–15·3)
Literacy in women, %	78·8% (70–86)	67·3% (56–75)	82·3% (78–83)
Age, years	27·1 (25·4–29·2)	24·0 (22·6–26·6)	27·8 (25·2–29·7)
Total population older than 60 years, %	7·2% (5–9)	6·6% (6–8)	5·7% (4–6)
Households with clean cooking fuel, %	42·0% (24–66)	31·2% (17–47)	79·7% (72–89)
Gross domestic product per capita, 2017 USD	$4251 (2654–6355)	$3273 (1620–7217)	$5845 (3533–7277)
Temperature from December to February,† °C	21·8 (15·7–24·1)	17·7 (15·8–19·3)	15·6 (14·6–16·3)
Temperature from March to May,† °C	27·9 (22·6–30·1)	29·5 (28·3–30·6)	28·9 (28·1–29·4)
Temperature from June to August,† °C	26·8 (24·5–28·6)	29·9 (28·9–31·1)	31·3 (30·7–31·7)
Temperature from September to November,† °C	25·2 (22·3–26·6)	25·6 (24·8–26·2)	25·5 (24·9–25·9)
Completeness of death registration, %	88·8% (60–100)	70·1% (52–92)	88·6% (46–100)
Annual PM_2·5_,† μg/m^3^	28·5 (23·4–34·4)	50·4 (44·5–59·9)	96·5 (86·8–99·4)

Data median (IQR) unless otherwise specified.

* In total there are 28 states and eight union territories, and 136 divisions; some states, union territories, and divisions fall under more than one percentile group.

† Population-weighted mean (IQR).

**Table 2:** Attributable deaths and fractions

	Attributable deaths, millions (95% CI)	Attributable fraction, % (95% CI)

**PM_2·5_ concentrations higher than Indian guidelines (≤40 μg/m^3^)**		
Total (11-year period)	3·8 (2·9–4·9)	5·0% (3·8–6·4)
Mean deaths per year	0·3 (0·2–0·4)	5·0% (3·8–6·4)
**PM_2·5_ concentrations higher than WHO guidelines (≤5 μg/m^3^)**		
Total (11-year period)	16·6 (13·0–21·8)	24·9% (19·5–32·5)
Mean deaths per year	1·5 (1·1–1·9)	24·9% (19·5–32·5)

## Data Availability

All the data in this study are routinely collected and contain no information about specific people. Our data are available on request to the corresponding author, subject to the agreement of the CHAIR-India Publications and Executive Committee.

## Research in context

### Evidence before this study

The evidence supporting the association between long-term exposure to ambient air pollution and mortality in India is scarce and inconsistent with studies in other countries. We searched PubMed on April 10, 2024, for literature published within the past 10 years (since April 10, 2014) in English on long-term exposure to PM_2·5_ and all-cause mortality in India using the search terms “ambient air pollution”, “ambient particulate matter”, “particulate matter”, “long-term effects”, “mortality”, and “India”. There were few studies from India examining the association of cause-specific and premature mortality and ambient PM_2*·*5_ exposure by use of the framework of health impact assessment and exposure–response function from the Global Burden of Diseases, Injuries, and Risk Factors Study. These studies did not apply any causal inference methods and PM_2*·*5_ exposures from coarser exposure assessment than our spatiotemporal model. One publication examined the association of long-term exposure to PM_2·5_ and all-cause mortality in India but included only a small fraction of all deaths occurring in India in the studied period and did not apply causal methods.

### Added value of this study

Using a quasi-experimental design, the difference-in-differences approach, our study provides a comprehensive and robust estimate of the association between long-term exposure to PM_2·5_ and mortality in India from 2009 to 2019, using a high-resolution spatiotemporal model for PM_2·5_ coupled with fine-scale population data and district-level mortality data. We included all of the deaths registered in the country across the study period. Our estimates show harmful effects of PM_2·5_ well below the India National Ambient Air Quality Standards for annual PM_2·5_ exposure.

### Implication of all the available evidence

The extremely high levels of air pollution in India contribute to a higher fraction of mortality than previously estimated, with high mortality even at concentrations below the current Indian national air quality standards. This study, and the available evidence from other studies across the world, strongly underpins the need for India to provide appropriate regulation to reduce emissions and protect public health.
