# Supplementary material for: Estimating the effect of annual PM2·5 exposure on mortality in India: a difference-in-differences approach
Source: Lancet Planet Health. Author manuscript; Available in PMC 2025 Feb 3. (PMC11790315; doi:10.1016/S2542-5196(24)00248-1)
Supplement: Supplement file [file NIHMS2045242-supplement-Supplement_file.pdf]

THE LANCET
Planetary Health

Supplementary appendix

This appendix formed part of the original submission and has been peer reviewed. We post it as supplied by the authors.

Supplement to: Jaganathan S, Stafoggia M, Rajiva A, et al. Estimating the effect of annual PM2·5 exposure on mortality in India: a difference-in-differences approach. *Lancet Planet Health* 2024; **8:** e987–96.

Supplementary material

Estimating the effect of annual $PM_{2.5}$ exposure on mortality in India: A Difference-in-Differences Approach

Authors: Suganthi Jaganathan[1,2,3] MPH, Massimo Stafoggia[1,4] PhD, Ajit Rajiva[2,3,5] MESc, Siddhartha Mandal[2,3] PhD, Shweta Dixit[2,3] PhD, Jeroen de Bont[1] PhD, Prof Gregory A. Wellenius[6] ScD, Kevin J. Lane[6] PhD, Amruta Nori-Sarma[6] PhD, Prof Itai Kloog[5,7] PhD, Prof Dorairaj Prabhakaran[3,8] DM, Poornima Prabhakaran[2,3]# PhD, Prof Joel Schwartz[9]# PhD, Petter Ljungman[1,10]# PhD

#Shared last authors

Affiliations:
1-Institute of Environmental Medicine, Karolinska Institutet, Stockholm, Sweden
2- Centre for Health Analytics Research and Trends, Ashoka University, Sonipat, Haryana, India
3-Centre for Chronic Disease Control, Delhi-NCR, India
4-Department of Epidemiology, Lazio Region Health Service / ASL Roma 1, Rome, Italy
5-Department of Geography and Environment, Faculty of Humanities and Social Sciences, Ben-Gurion University of the Negev, Beer-Sheva, Israel
6-Department of Environmental Health, School of Public Health, Boston University, Boston, MA, USA
7- Department of Environmental Medicine and Public Health, Icahn School of Medicine at Mount Sinai, New York, NY, USA
8-Public Health Foundation of India, Gurugram, Haryana, India
9-Department of Environmental Health, Harvard T.H. Chan School of Public Health, Boston, MA, USA
10-Department of Cardiology, Danderyd University Hospital, 182 57, Danderyd, Sweden

Corresponding author:

Email: suganthi.jaganathan@ki.se
Address: Institute for Environmental Medicine
Karolinska Institutet
SE-171 76 Stockholm
Sweden

Table of contents:

Figure S1: Directed Acyclic Graph (DAG) for the covariates used in the analyses.
Table S1: Spatiotemporal data availability of covariates.
Table S2: Steps in the DID model development along with model specifications
Table S3: Results from the sensitivity analyses.
Table S4: Attributable deaths (n) & fraction (%) per year by $PM_{2.5}$ for levels higher than Indian guidelines 40 μg/m^3 (95% CI) & WHO guidelines 5 μg/m^3 (95% CI).

Figure S1: Directed Acyclic Graph (DAG) for the covariates [District-level] used in the analyses.

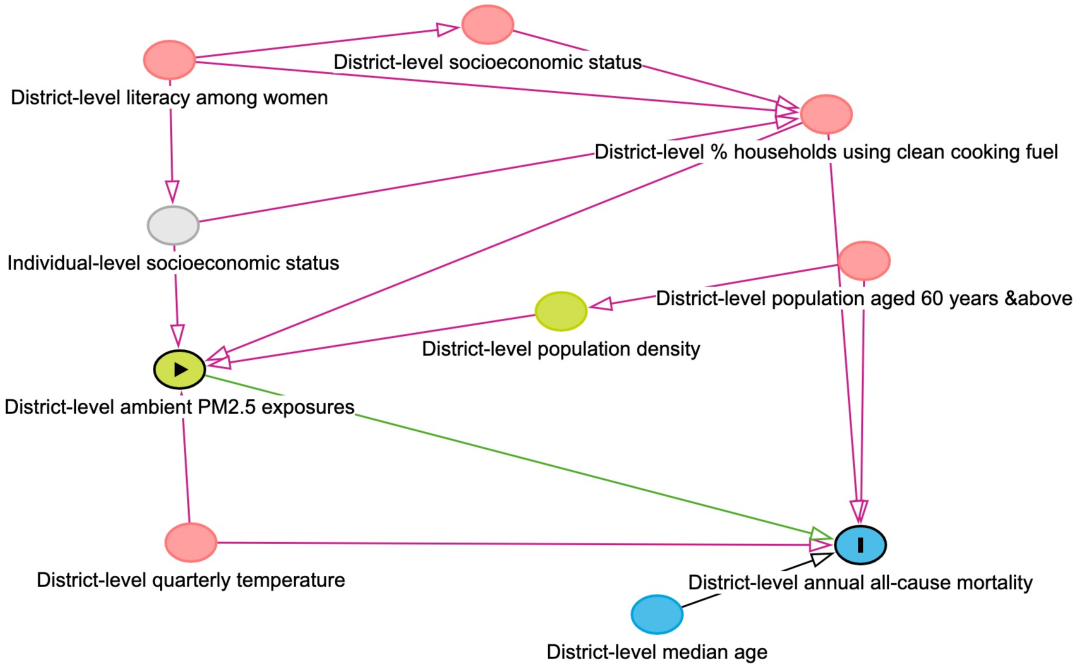

*PM2·5 exposures were population-weighted averages

Table S1: Spatiotemporal data availability of covariates

S.no.	Variable description	Indicator	Spatial resolution	Temporal resolution	Time period	Source
**I. Variables available at 1 sq·km grid level**						
1.	$PM_{2·5}$	Population weighted mean	District-level	Annual	2009 to 2019	CHAIR India spatiotemporal model[1]
2.	Temperature	Population weighted mean	District-level	Quarterly	2009 to 2019	European Centre for Medium Range Weather Forecast (ECMWF)
3.	Gross Domestic Product (GDP)	GDP percapita	District-level	Annual	2009 to 2019	Chen et al 2022[2]
4.	Population density	Gridded population density	District-level	Annual	2010, 2015 & 2020	Center for International Earth Science Information Network (CIESIN) - Columbia University, Socioeconomic Data and Applications Center (SEDAC)[3]
**II. Variables available at district level**						

1.	All-cause mortality	All age groups	District-level	Annual	2009 to 2019	Registrar General, Ministry of Home Affairs, Vital Statistics Division, Civil Registration System (CRS) Section[4]
2.	Population above 60 years of age	Proportion of population aged 60 and above per 10000	District-level	Annual	2011	Census 2011[5]
3.	Clean cooking fuel	Proportion of households using clean fuel for cooking	District-level	Annual	• 2011<br>• 2014-2015 and<br>• 2019-2020	Census 2011[5], National Family Health Survey (NFHS)[6,7]: Wave 4 (2014-2015) & Wave 5 (2019-2020)
4.	Literacy among women	Proportion of literate women	District-level	Annual	• 2014-2015 and<br>• 2019-2020	National Family Health Survey (NFHS)[6,7]: Wave 4 (2014-2015) & Wave 5 (2019-2020)
**III. Variables available at state level**						
1.	Median age	Median age	State-level	Annual	2011, 2016 and 2021	National Commission on Population, 2020[8]
2.	Death Registration levels	Proportion of completeness of death registration level	State-level	Annual	2009 to 2019	Registrar General, Ministry of Home Affairs, Vital Statistics Division, Civil Registration System (CRS) Section[4]

**Table S2: Steps in the DID model development along with model specifications**		
**Details of the model**	**Equation**	**Increased risk % per 10 µg/m^3 (95%CI)**
Model 1: (crude model)	Y~ $PM_{2·5}$	14·1 (11·8 -16·5)
Model 2: (adjusted for observed confounders)	Y~ $PM_{2·5}$+ covariates	6·8 (4·7 -9·0)
Model 3: [only temporal adjustment]	Y~ $PM_{2·5}$+ covariates + I(years)	9·4 (7·2-11·7)
Model 4a & b: [only spatial adjustment]	Y~ $PM_{2·5}$+ covariates + I(states)	5·8 (4·0-7·7)
	Y~ $PM_{2·5}$+ covariates + I(divisions)	6·8 (4·7-8·9)
Model 5: TWFE [two-way fixed effects]	Y~ $PM_{2·5}$+ covariates + I(years) + I(divisions).	9·4 (7·3-11·7)
Final Model: IFE [interactive fixed effects] [DID Model]	Y~ $PM_{2·5}$+ covariates + ns(trend)*I(divisions)	8·6 (6·4-10·8)

**Table S3: Results from the sensitivity analyses**		
**Details of the model**	**Model specifications**	**Increased risk % per 10 µg/m^3 (95%CI)**
Model accounted for variations in completeness of death reporting	Y= (Mortality rate * percentage of completeness of death reporting) used in the final model	7·5 (5·3-9·8)
To check robustness of the results from the final model	Excluding data from districts with < 1st and >99th percentiles of $PM_{2·5}$	10·5 (8·1-13·0)

Table S4: Attributable deaths (n) & fraction (%) per year by $PM_{2\cdot5}$ for levels higher than Indian guidelines 40 µg/m^3 (95% CI) & WHO guidelines 5 µg/m^3 (95% CI)				
**Year**	**Attributable deaths, n in millions [India] 95% CI**	**Attributable deaths, n in millions [WHO] 95% CI**	**Attributable fraction (%) [India] 95% CI**	**Attributable fraction (%) [WHO] 95% CI**
**2009**	0·2 (0·1-0·2)	1·1 (0·9-1·5)	4·4 (3·3-5·6)	24·5 (19·2-32·0)
**2010**	0·2 (0·1-0·3)	1·2 (0·9-1·5)	4·5 (3·4-5·8)	24·4 (19·1-31·8)
**2011**	0·2 (0·1-0·2)	1·1 (0·9-1·5)	3·3 (2·5-4·2)	23·3 (18·2-30·3)
**2012**	0·2 (0·1-0·3)	1·2 (0·9-1·6)	3·8 (2·9-4·9)	24·1 (18·9-31·4)
**2013**	0·4 (0·3-0·5)	1·6 (1·3-2·2)	6·2 (4·8-8·0)	26·3 (20·7-34·5)
**2014**	0·3 (0·3-0·5)	1·6 (1·2-2·1)	5·5 (4·2-7·0)	25·5 (20·0-33·3)
**2015**	0·3 (0·3-0·5)	1·5 (1·2-2·0)	5·6 (4·3-7·1)	25·4 (19·9-33·2)
**2016**	0·4 (0·3-0·5)	1·5 (1·2-2·0)	5·8 (4·5-7·5)	25·9 (20·3-33·9)
**2017**	0·4 (0·3-0·5)	1·6 (1·3-2·2)	5·5 (4·2-7·1)	25·3 (19·8-33·1)
**2018**	0·4 (0·3-0·6)	1·8 (1·4-2·4)	5·6 (4·3-7·2)	25·5 (20·0-33·4)
**2019**	0·3 (0·2-0·4)	1·8 (1·4-2·4)	3·8 (2·9-4·8)	23·0 (18·0-30·0)

References:

1. Mandal S, Rajiva A, Kloog I, Menon JS, Lane KJ, Amini H, et al. Nationwide estimation of daily ambient PM2.5 from 2008 to 2020 at 1sq.km. in India using an ensemble approach. PNAS Nexus. 2024 Feb 27;
2. Chen J, Gao M, Cheng S, Hou W, Song M, Liu X, et al. Global 1 km × 1 km gridded revised real gross domestic product and electricity consumption during 1992–2019 based on calibrated nighttime light data. Sci Data. 2022 May 12;9(1):202.
3. Center for International Earth Science Information Network - CIESIN - Columbia University. 2018. Gridded Population of the World, Version 4 (GPWv4): Population Density, Revision 11. Palisades, NY: NASA Socioeconomic Data and Applications Center (SEDAC). https://doi.org/10.7927/H49C6VHW. Accessed 2023 Jun 25.
4. Office of the Registrar General of India, Ministry of Home Affairs, Government of India. Vital statistics of India based on the civil registration system. [Internet]. https://censusindia.gov.in/census.website/data/VSREPORT. [cited 2023 Jun 25]. Available from: https://censusindia.gov.in/census.website/data/VSREPORT.
5. Census of India (2011). Office of the Registrar General & Census Commissioner, New Delhi [Internet]. [cited 2023 Jun 25]. Available from: https://censusindia.gov.in/census.website/data/census-tables
6. International Institute for Population Sciences (IIPS) and ICF. 2017. National Family Health Survey (NFHS-4), 2015-16: India. Mumbai: IIPS.
7. International Institute for Population Sciences (IIPS) and ICF. 2021. National Family Health Survey (NFHS-5), India, 2019-21: Mizoram. Mumbai: IIPS .
8. National Commission on Population (2020). Population Projections for India and States 2011-2036: Report of the Technical Group on Population Projections. National Commission on Population, Ministry of Health & Family Welfare [Internet]. [cited 2023 Jun 25]. Available from: https://main.mohfw.gov.in/sites/default/files/Population%20Projection%20Report%202011-2036%20-%20upload_compressed_0.pdf
